# Image-Guided Robotic Stereotactic Radiation Therapy with Fiducial-Free Tumor Tracking for Lung Cancer

**DOI:** 10.1186/1748-717X-7-102

**Published:** 2012-06-24

**Authors:** Jean-Emmanuel Bibault, Bernard Prevost, Eric Dansin, Xavier Mirabel, Thomas Lacornerie, Eric Lartigau

**Affiliations:** 1 Academic Radiation Therapy Department, Lille II-Nord de France University, CyberKnife Nord-Ouest, Oscar Lambret Comprehensive Cancer Center, 3, rue Frédéric Combemale, BP 307-59020, LILLE Cedex, France; 2 General Oncology Department, Oscar Lambret Comprehensive Cancer Center, 3, rue Frédéric Combemale, BP 307-59020, LILLE Cedex, France

**Keywords:** CyberKnife, Hypofractionated, Robotic Stereotactic Body Radiation Therapy, Lung Cancer, Xsight Lung, Efficacy, Toxicity

## Abstract

**Purpose:**

Stereotactic body radiation therapy (SBRT) for early-stage lung cancer can be achieved with several methods: respiratory gating, body frame, or real-time target and motion tracking. Two target tracking methods are currently available with the CyberKnife® System: the first one, fiducial tracking, requires the use of radio-opaque markers implanted near or inside the tumor, while the other, Xsight® Lung Tracking System, (XLTS) is fiducial-free. With XLTS, targeting is synchronized directly with target motion, which occurs due to respiration. While the former method (fiducial tracking) is well documented, the clinical relevance of the latter (tracking without fiducials) has never been well described to this date.

**Patients and Methods:**

A study was performed at our department for each patient treated for lung cancer with CyberKnife using XLTS. Selection criteria were: primary or recurring T1 or T2 stage non-small-cell lung cancer (NSCLC) with 15–60 mm tumor size. Initial staging included CT-Scan and FDG-PET.

**Results:**

Fifty-one patients not amenable to surgery were treated with XLTS. Median follow-up was 15 months (range, 5–30 months). Median tumor size was 24 mm (range, 15–60 mm). Median total dose was 60 Gy (36–60 Gy) in three fractions. Actuarial overall survival was 85.5% (95% CI = 74.5–96%) at 1 year and 79.4% (95% CI = 64–94.8%) at 2 years. Actuarial local control rate was 92% (95% CI = 84–99%) at one1 year and 86% (95% CI = 75–97%) at 2 years.

**Conclusion:**

Local control and overall survival rates were similar to previous reports that used fiducials for tumor tracking. Toxicity was lower than most studies since tumor tracking did not require fiducial implantion. This fiducial-free method for respiratory motion tracking is a valid option for the most fragile patients.

## Introduction

Stereotactic Body Radiation Therapy (SBRT) use is rapidly increasing among patients with lung cancer not amenable to surgery. Several methods are currently available suitable for the delivery of such high doses to small volumes. The required precision can be attained through several techniques, one of which involves tracking the tumor’s movement in real time while the patient breathes freely.

The CyberKnife® system (Accuray Incorporated, Sunnyvale, California, USA) was first introduced in France in 2006 through the financial support of the French National Cancer Institute (INCa). It allows for stereotactic body radiation therapy (SBRT) of lung cancer with real-time target and motion tracking. Two target tracking methods are currently available with the system: one of them, Fiducial Tracking, requires the use of fiducial markers implanted near or inside the tumor, while the other, Xsight® Lung Tracking System, (XLTS) is fiducial-free. Both tumor tracking methods can be combined with the Synchrony® Respiratory Tracking System, which synchronizes the beam targeting during delivery with the motion of the target due to respiration. While the former (Fiducial Tracking with Synchrony) is well documented, the efficacy and toxicity of the latter (XLTS with Synchrony) has not been well described.

Transthoracic fiducial implantation is reportedly responsible for cases of pneumothorax in 13% of the patients that undergo the procedure. [1] This rate may actually be an underestimate as some studies have reported rates of 23%, or even 38%, for transthoracic biopsies [2-5]. Considering that the concerned population of patients is often very fragile (elderly patients, those with chronic lung disease or other malignancies), the risk of pneumothorax should not be taken lightly. Patients are sometimes excluded only because they could not afford the risk of a pneumothorax. Fortunately, other fiducial implantation techniques are available, such as electromagnetic navigation-guided bronchoscopy or intravascular coil placement. However, the use of radiomarkers has others risks beyond pneumothorax, such as arrhythmias in the case of endovascular coils. They must be inserted with high precision for the tracking system to work, and sometimes they migrate and cause a systematic error at each treatment session. Finally, fiducial insertion may delay the treatment, since it is better to perform the planning CT a few days after the insertion.

With these difficulties in mind, a new system has been developed to directly track the tumor instead fiducials. XLTS is able to correlate intensity similarities in the digitally reconstructed radiographs (DRRs) to the position of the tumor when certain tumor criteria are met. This method may represent a shorter, completely uninvasive treatment for patients with lung cancer not amenable to surgery. In this study, we discussed the technical aspects of this system, the precise patient selection criteria required for its application, and the clinical outcome in terms of both efficacy and toxicity for the 51 patients treated at our center between November 2008 and January 2011.

## Patients and Methods

### CyberKnife and Xsight Lung Tracking System

**Figure 1:**
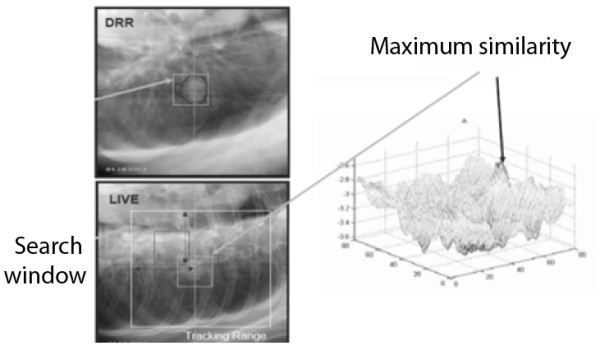
Upper-left corner : treatment planning DRR; Lower-left corner: Live imaging from the CyberKnife; Right image: System searching for the maximum similarity between DRR and live.

Pulmonary tumors larger than 15 mm and located in the peripheral or apex regions are visible in the orthogonal X-Ray images created by the CyberKnife System. Direct tumor tracking is accomplished by matching the image intensity pattern of the tumor region in the digitally reconstructed radiographs (DRRs) to the corresponding region in the treatment X-ray images (Figure 1). A correlation model is then generated by fitting the internal tumor positions at different phases of the breathing cycle to the simultaneous external marker positions. During treatment, the internal tumor position is estimated from the external marker positions using the correlation model. The beam is moved dynamically with the target in order to maintain alignment of each treatment beam in real time. Phantom experiments have showed that the total system error is 1.07 mm when XLTS is used for tracking tumors [6-10].

### Patient eligibility

Patient inclusion criteria were a single primary or recurring pulmonary lesion, T1 or T2 stage with a tumor diameter between 15 and 60 mm without lymph node involvement or distant metastases, treated from November 1, 2008, to January 1, 2011. Initial staging included CT-Scan (thoracic, abdominal, and pelvic) with ^18^ F-fluorodeoxyglucose positron emission tomography (FDG-PET). Histological proof was obtained using transthoracic or bronchoscopic biopsy. If histological proof could not be obtained, patients were treated when the lesion was considered evolutive, i.e., increasing in size on two consecutive CT-Scans with uptake of FDG on a single PET. Any lung-infectious process, particularly tuberculosis, was searched and ruled out before treatment. Patients had a Performance Status under 2. Patients with a history of other neoplasm were excluded. Each case was discussed during our thoracic oncology department staff meeting by a radiation oncologist, a medical oncologist, a radiologist, and a thoracic surgeon. Lesions were considered surgically removable in patients not amenable to surgery. Main inoperability reasons included pre-existing medical conditions such as advanced chronic obstructive pulmonary disease (GOLD 3, predicted FEV_1_ < 49%), cardiovascular disease (ejection fraction < 40%), or general anaesthesia contraindications such as obesity (BMI > 30 kg/m^2^) or an history of anaphylactic shock to the anaesthesia. Previous contra or ipsilateral lung surgery or radiation therapy was allowed. Patients did not receive chemotherapy before, during, or after treatment until any progression. Written informed consent was obtained from each patient.

Certain tumor characteristics were required for XLTS to be able to detect the tumor: the tumor had to be at least 15 mm in diameter, inside the lung parenchyma, and at least 15 mm from any major vascular structure or ribs. The projection of the tumor on the spine could not be at a 45° angle because of the CyberKnife’s X-Ray tube angulation.

### Treatment planning, dose calculation, and treatment session

A thin-sliced CT-scan without contrast was recorded with millimetric slices. The gross tumor volume (GTV) and organs at risk (spinal cord, left and right lung, heart, and esophagus) were contoured on CT-Scan with the following window and level setting: -600/1600 HU. A geometrical 3-mm margin was added to the GTV to create the planning target volume (PTV). Treatments were planned on Accuray's Multiplan® software. The dose was calculated using the Ray-Tracing algorithm. Dose was prescribed on the 83% isodose line. Dose constraints were as follow: total lung volume (defined as right and left lung excluding PTV) receiving 5 Gy <50% (V5 <50%), and V10 <30%; for heart, V24 was <15 cm^3^ and maximal dose <30 Gy; for trachea and bronchi, V15 was <4 cm^3^ and V20 <1 cm^3^, with maximum acceptable point doses at 30 Gy. Maximum dose to esophagus was 25 Gy and V21 was <5 cm^3^.

### Study endpoints

The XLTS has already been validated on phantom experiments^13-17^, but its clinical validity has not been reported yet. The primary objective of this study was to evaluate the local control rate achievable with XLTS. The secondary objective was to evaluate the toxicity. Local control was calculated from the time of treatment until tumor relapse within PTV. Patients without local relapse were censored on the day of the last follow-up. Local relapse was defined as a 20% increase of the maximum diameter of the tumor on CT-Scan compared to initial staging. Overall survival was calculated from the start of radiotherapy until death from any cause. Disease-specific survival was measured from the start of radiotherapy until death from lung cancer. Patients alive at last follow-up were censored. Follow-ups included a CT-Scan at 3, 6, 9, and 12 months after treatment and every 6 months after that; and an FDG-PET every 6 months. Treatment response was evaluated according to RECIST (Response Evaluation Criteria In Solid Tumors) v1.1 [11]. Toxicity was evaluated according to CTCAE (National Cancer Institute Common Terminology Criteria for Adverse Events) v4.0.

### Statistics

SPSS (SPSS Inc., Chicago, Illinois, USA) version 13 software was used for statistical analyses. Kaplan-Meier method with 95% confidence intervals was used to estimate local, overall, and specific survival. Cox regression analysis was used to find prognostic values of patient and tumor characteristics on local control and survival endpoints. Differences between groups were illustrated with Kaplan-Meier curves and the log-rank test. All tests were two-sided. A significance level α = 0.05 was used.

## Results

### Patient characteristics

Fifty-one patients were treated for non-small-cell lung cancer with XLTS between November 1, 2008, and January 1, 2011. None of the patients were eligible for surgery. Forty-three (84%) patients were men and eight (16%) were women. Median age was 69 years (range, 50–85 years). All patients were smokers who had quit at time of treatment. Histology was known for 19 patients (38%): 10 were squamous-cell carcinomas (20%), six adenocarcinomas (12%), and three undifferenciated cancers (6%). Thirty-one patients had T1 tumors and 20 had T2. Median tumor size was 24 mm (range, 15–60 mm). Eight patients were treated for a recurrence after prior surgery (n = 5) or radiation therapy (n = 3). All 51 patients had FDG-PET before treatment. The treatment obviated the necessity to implant fiducials along with its common sequelae such as pneuthorax. Main characteristics are presented in Table 1.

**Table 1:** Patient characteristics

			**Men**	**Women**
**Sex**			43 (84%)	8 (16%)
		**Median**	**Minimum**	**Maximum**
**Tumor Size (mm)**		24	11	60
**Age (years)**		68	50	85
	**Adenocarcinoma**	**Squamous Cell Carcinoma**	**Undifferenciated**	**Unknown**
**Histology**	10 (20%)	6 (10%)	3 (5%)	16 (62%)

### Technical characteristics of performed treatments

Median delivered dose was 60 Gy (range, 45–60 Gy). Three fractions were performed for the treatment, but if the tumor was considered too close to mediastinal structures, the dose/fraction was lowered to 15 Gy and four fractions were performed. This occurred in the case of three patients. Median GTV was 11 cm^3^ (range, 1.6–115 cm^3^) and median PTV was 25 cm^3^ (range, 4–142 cm^3^). Median number of beams used was 70 (range, 20–163 beams), and median treatment session duration was 63 minutes (range, 27–134 minutes). The dose was prescribed to the 84% isodose line. Treatment characteristics are presented in Table 2.

**Table 2:** Technical characteristics of the treatments

	**Median**	**Minimum**	**Maximum**
**GTV (cm**^**3**^**)**	12	1.6	115
**PTV (cm**^**3**^**)**	25	4	142
	**Median**	**Minimum**	**Maximum**
**Total Dose (Gy)**	60	45	60
**Dose/Fraction (Gy)**	20	15	20
**Number of treatment sessions**	3	3	4
**Duration of the sessions (min)**	63	27	134
**Number of treatment beams**	70	20	163

### Treatment response

**Figure 2:**
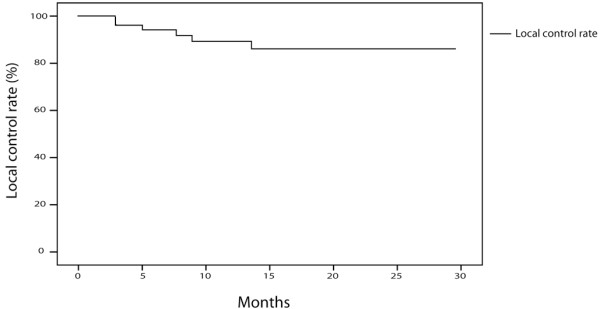
Local control rates of patients treated with the Xsight Lung Tracking System (n = 51).

Median follow-up was 15 months (range, 5–30 months). Actuarial local control was 92% (95% CI = 84–99%) at 1 year and 86% (95% CI = 75–97%) at 2 years (Figure 2). Five patients (10%) experienced local failure along with distant metastases to bone (n = 3), liver (n = 1), brain (n = 2), or controlateral lung (n = 2). One patient’s response to the treatment could not be evaluated on the first CT-Scan at three months because of radiation pneumonitis surrounding the treated tumor. However, evaluation at 12 months showed a complete resolution of the pneumonitis and a partial response. One patient relapsed with mediastinal lymph node metastases two years after treatment.

Actuarial overall survival was 85.5% (95% CI = 74.5–96%) at 1 year and 79.4% (95% CI = 64–94.8%) at 2 years. Seven patients died during follow-up, five from cancer progression and two due to intercurrent disease. Actuarial disease-specific survival was 90.1% (95% CI = 71–100%) at 1 year and 84% (95% CI = 69–98%) at 2 years.

### Prognostic factors for overall survival and local control

**Figure 3:**
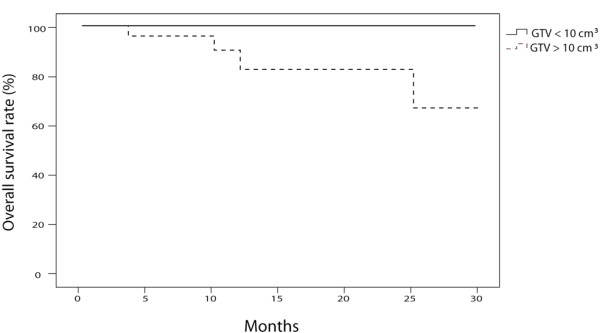
**Overall survival of patients with GTV > 10 cm**^**3**^**(n = 23) and patients with GTV < 10 cm**^**3**^**(n = 28) treated with the Xsight Lung Tracking System (p = 0.029).**

Patients, tumor and treatment characteristics were tested as prognostic factors for overall survival and local control. With regard to overall survival, no significant difference based on sex (p = 0.254), age (p = 0.512), availability of histologic data (p = 0.190), total dose delivered (p = 0.705), or number of fractions (p = 0.543) was found. Patients with a maximum tumor diameter under 3 cm tended to have a better overall survival (p = 0.052). GTV greater than 10 cm^3^ was significantly associated with poorer survival (100% vs. 65.2% at 2 years, p = 0.029, Figure 3).

**Figure 4:**
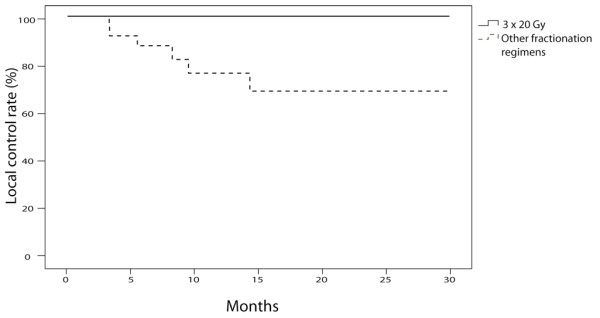
Local control rates of patients treated with three fractions (n = 27) and patients treated with more than three fractions (n = 24) with Xsight Lung Tracking System (p = 0.006).

Regarding local control, no significant difference was observed based on sex (p = 0.886), age (p = 0.26), availability of histologic data (p = 0.98), tumor size (p = 0.224), GTV (p = 0.171) or total dose delivered (p = 0.33). A significant difference *was* found between patients treated with three fractions and patients treated with more than three fractions: the local control rate at 2 years was 100% for patients treated with three fractions and 70% for patients treated with more than three fractions (p = 0.006, Figure 4).

### Toxicity

No pneumothorax was observed as we did not need fiducials for tumor tracking. Seven cases of grade 1 radiation pneumonitis (14%) without any clinical impact and one grade 2 (2%) radiation pneumonitis were observed at 3 months. No steroids were required for any of these patients. Three grade 1 (6%) radiation fibroses were observed on CT-Scan performed 1 year after treatment. No acute or late grade 3 or 4 toxicities were observed.

## Discussion

Several studies concerning SBRT for early-stage lung cancer have been published. After White et al. published a dose-escalation study in 2003, series reporting about patients treated with CyberKnife began to emerge. In 2006, Nuyttens et al. wrote about 20 patients treated for 22 lung tumors [12]. The chosen method of treatment required the use of fiducials. The reseachers used 78 markers to track the tumors: 34 were implanted using transthoracic punction, 23 were endovascular coils, and 21 were extrathoracic. This study reported no pneumothorax. Local control rate was 100% at four months. In 2007, Collins et al. published a study about 25 patients also treated using fiducials [13]. Seven of the 25 patients presented with pneumothorax. Another study published in 2008 by Castelli *et al.* about 30 patients showed four cases of pneumothorax and one fiducial migration [14]. The study with the most patients was published in 2009 by van der Voort van Zyp et al. [15]: 70 surgery-ineligible patients (39 with T1 tumors and 31 with T2 tumors) were treated with 45 Gy or 60 Gy in three fractions. Local control rate was 96% for patients treated with 60 Gy and 78% for patients treated with 45 Gy. A total of 225 fiducials were used (143 endovascular coils, 72 implanted using bronchoscopy, and 10 implanted by transthoracic punction). One to five fiducials were used for each patient for tumor tracking. Fiducial implantation induced two grade 3 toxicities (one pneumothorax requiring chest drain and an arrhythmia after intravascular coil placement), one grade 2 toxicity (pneumothorax), and six grade 1 (dyspnea n = 1, pneumothorax n = 2, and self-limiting hemorrhage, n = 3). These toxicities are not frequent but cannot be neglected.

Our study is the first, to our knowledge to report only on patients who were treated with a fiducial-free method for tumor tracking. We report a local control rate of 88% at 15 months, which is comparable to other studies, which indicates the method could be used without losing efficacy. However, treatment time of over 60 minutes can potentially be associated with loss of tumor BED of > 10–15%, which will impact tumor control significantly^15^. Median treatment time for our patient was 63 minutes (range, 27–134 minutes). This may explain our local failure rate. Therefore, the number of pencil beams should be kept to a minimum to decrease the treatment time in cyberknife treatments.

Patients treated with 4 or more fractions had a worse local control rate than patients treated in three fractions. This could be explained by the lower BED resulting from the increased number of sessions for the same total dose (BED for 60 Gy/4fx = 150 Gy vs BED for 60 Gy/3fx = 180 Gy). The number of treatment session should therefore be kept to three.

Five patients (10%) with local control relapsed with distant metastases to bone (n = 3), liver (n = 1), brain (n = 2), or the controlateral lung (n = 2). One patient relapsed with mediastinal lymph node metastases 2 years after treatment. A major limitation of our study was the small number of patients with known histology (38%). This is often the case in SBRT studies for lung cancer. Van der Voort van Zyp et al. have reported an absence of confirmation of malignancy in 49% of the patients. Most of the patients are treated with SBRT because they are too fragile for surgery or even trans-thoracic biopsies. However, Swensen et al. have created a malignancy prediction model that, combined with FDG-PET [16], could be useful for that matter.

We report seven grade 1 (14%) and two grade 2 (14%) radiation pneumonitis cases and no grade 3 or 4 incidents. These rates are lower than those that have been reported by others (3–10% grade 3 toxicity) [17-19]. No patient had any post-treatment pain or rib fractures. This may be due to our strict selection criteria for XLTS, which mandated that the tumors be at least 15 mm away from the ribs. We observed three cases of grade 1 lung fibrosis.

There is an important need for multicenter randomized trials comparing surgery to SBRT. An international randomized prospective trial (STARS, ClinicalTrials.gov ID: NCT00840749) was initiated by Accuray in collaboration with the MD Anderson Cancer Center (Texas, USA) in December 2008 to compare surgery to SBRT for operable patients with early-stage non-small-cell lung cancer tumors under 4 cm in diameter without lymph node or distant metastases. Possible surgeries include: lobectomy, bilobectomy, or pneumonectomy. The primary endpoint is overall survival. On the other hand, the European multicenter randomized trial (ROSEL, ClinicalTrials.gov ID: NCT00687986) comparing SBRT to surgery for operable patients has recently been terminated due to poor recruitment.

## Conclusion

Lung SBRT with fiducial-free tumor tracking is both feasible and effective. Local control rate is similar to what so far has been reported in other studies that have used markers for tumor tracking. Toxicity was lower as there was no fiducial implantation, thereby preventing cases of pneumothorax normally associated with this procedure. However, precise patient selection according to simple criteria was required for the Xsight Lung System to work. This system is an interesting treatment option for patients not amenable to surgery or too fragile for transthoracic punction and fiducial implantation. A standard 3 x 20-Gy fractionation regimen should be used to achieve optimal local control.

## Competing interests

No conflict of interest to declare.

## Authors’ contributions

JE, BP and EL conceived the study. JE collected data and drafted the manuscript. BP, ED, XM, TL and EL participated in coordination and helped to draft the manuscript. JE performed the statistical analyses. EL provided mentorship and edited the manuscript. All authors have read and approved the final manuscript.

## Summary

SBRT is a growing field in the management of early-stage lung cancer. Several methods are available to achieve precision: respiratory gating, body frame, or real-time tumor tracking. Real-time tumor tracking can be performed with fiducials or with a new method that tracks the tumor itself. We report the first clinical study using this fiducial-free treatment. With local control rates of 92% and 86% at 1 and 2 years, respectively, the results of this method seem comparable to other studies that use fiducials.
